# Incorporation of Encapsulated Omega-3 in 3D-Printed Food Gels: A Study on Rheology, Extrusion, and Print Performance in Dual Ink Printing

**DOI:** 10.3390/foods14152681

**Published:** 2025-07-30

**Authors:** Adrián Matas-Gil, Francisco de-la-Haba, Marta Igual, Purificación García-Segovia, Javier Martínez-Monzó

**Affiliations:** 1 I-FOOD, Instituto Universitario de Ingeniería de Alimentos-FoodUPV, Universitat Politècnica de València, 46022 Valencia, Spain; admagi@doctor.upv.es (A.M.-G.); marigra@upvnet.upv.es (M.I.); pugarse@tal.upv.es (P.G.-S.); 2 Research Institute of Meat and Meat Products (IProCar), University of Extremadura (UNEX), 10003 Cáceres, Spain; frandelahaba@unex.es

**Keywords:** 3D food printing, functional foods, omega-3 encapsulation, pea protein, food gels, printability, extrusion, buildability

## Abstract

The integration of functional ingredients into 3D food printing formulations presents both opportunities and challenges, particularly regarding the printability and structural integrity of the final product. This study investigates the effect of incorporating omega-3 fatty acids encapsulated in pea protein into a model food gel composed of gelatin and iota-carrageenan. Four formulations with varying concentrations of encapsulated omega-3 (0%, 3%, 3.75%, and 6%) were evaluated for their rheological, textural, and printability properties. Rheological analysis revealed a progressive increase in storage modulus (G′) from 1200 Pa (0%) to 2000 Pa (6%), indicating enhanced elastic behavior. Extrusion analysis showed a reduction in maximum extrusion force from 325 N (0%) to 250 N (6%), and an increase in buffer time from 390 s to 500 s. Print fidelity at time 0 showed minimal deviation in the checkerboard geometry (area deviation: −12%), while the concentric cylinder showed the highest stability over 60 min (height deviation: 9%). These findings highlight the potential of using encapsulated bioactive compounds in 3D food printing to develop functional foods with tailored nutritional and mechanical properties.

## 1. Introduction

Three-dimensional food printing is an emerging technology that enables the transformation of digital designs into edible structures through additive manufacturing. Among the various techniques available, extrusion-based printing—either with or without thermal input—is the most widely used in food applications due to its simplicity and versatility [1]. This method has been successfully applied to a broad range of food matrices, including chocolate [2,3,4,5], doughs [6,7], purees [8,9,10,11], and protein-based gels [12,13,14].

As can be seen, this technique presents great versatility in food inks. The most outstanding feature is customization; it is possible to print food with different dimensions and shapes, filling structures, flavors, textures, etc. [15]. This customization, whether nutritionally or texturally, makes perfect tandem with the innovative trend of developing functional foods, such as for people with dysphagia [16,17,18,19].

Functional foods present modifications of incorporating or eliminating ingredients to benefit the consumer’s health [20]. Although eliminating ingredients such as lactose or gluten produces functional foods, the most widespread trend is the addition of ingredients with nutritional properties in foods [21,22]. It must be considered that due to internal conditions of the food or external conditions of processing, storage, or digestion, these functional ingredients degrade [23]. That is why they are often encapsulated by various materials, such as pea protein, to make them more resistant. By combining these two aspects of customization, 3D food printing and the incorporation of encapsulated functional ingredients, it is possible to develop functional foods where the featured ingredients deteriorate as little as possible. Some cases can be found in the literature where the food matrix itself acts as an encapsulant, but not in the addition of capsules to the food ink formulation [24,25,26].

The problem with adding any component to food inks is that they can change the rheological and textural properties and, therefore, the printability of the ink. Printability is the property of food ink to achieve a good printing process and result. This characteristic is divided into two concepts: extrudability, which is the ability of the ink to extrude well, and buildability, the ability to maintain the designed printed shape for an estimated time [1]. This is why many authors study these rheological and textural changes to try to predict or contrast the printability of new food inks [27,28,29,30].

This study aims to conduct a preliminary investigation of the impact on printability of incorporating omega-3 microcapsules into a model food gel composed of gelatin and iota-carrageenan. By evaluating rheological, textural, and printability properties across different capsule concentrations and geometries, the research seeks to identify only optimal conditions for producing functional 3D-printed foods with enhanced nutritional and structural characteristics.

## 2. Materials and Methods

For elaborating the gels, iota-carrageenan and pig gelatin from SOSA (Sosa Ingredients S.l.u, Navarcles, Spain) were used. Mineral water was purchased from a local supermarket. The encapsulated Omega-3 (fish oil) with pea protein (PP) was acquired through collaboration with Extremadura University (UEx, Cáreceres, Spain). The capsules were obtained by emulsifying water, fish oil, and pea protein and a subsequent spray-dryer process. The Omega-3 content as the sum of eicosapentaenoic acid (EPA) and docosahexaenoic acid (DHA) was 27 mg/g of capsule and with an average size of D[4, 3] = 14.46 μm and distributions of d(0.1)= 5.03 μm, d(0.5)= 11.51 μm, and d(0.9)= 23.12 μm measured with Mastersizer 2000 (Malvern Instruments Ltd. (Malvern, UK), Scirocco 2000).

### 2.1. Gels Elaboration

Four gel formulations were prepared using gelatin and iota-carrageenan as gelling agents, with varying concentrations of encapsulated omega-3 (0%, 3%, 3.75%, and 6% *w*/*w*). The base gel was composed of 5% gelatin and 2% iota-carrageenan, dissolved in water. The reason for the use of different formulations is explained later in Section 2.4.

To prepare the gels, 93 g and 100 g of mineral water were weighed and heated. The first one was heated to 60 °C and used to dissolve 5 g of gelatin, while the other was heated to 70 °C for 2 g of carrageenan dispersion. In formulations containing encapsulates, the capsules were added to the gelatin solution before reaching 60 °C to avoid thermal degradation. Both solutions were then combined, homogenized by magnetic stirring, and transferred into syringes (4.95 cm internal diameter, 1.13 cm internal diameter of tip, 10.63 cm length). The gels were allowed to rest at room temperature for 24 h before printing.

### 2.2. Rheology

Rheological measurements were performed using a rotational rheometer Rheostress 1 (Thermo Haake, Karlsruhe, Germany) equipped with a 60 mm parallel plate geometry. The linear viscoelastic region (LVR) was determined at 1 Hz over a stress range of 0.1–100 Pa. Frequency sweep tests were conducted at a constant stress of 1 Pa, ranging from 0.1 to 100 Hz. All analyses were carried out at room temperature (25 °C) and with a gap of 1 mm. The parameters recorded included storage modulus (G′), loss modulus (G″), complex modulus (G*), phase angle (tan δ), and apparent viscosity.

### 2.3. Extrusion Process Evaluation

**Figure 1:**
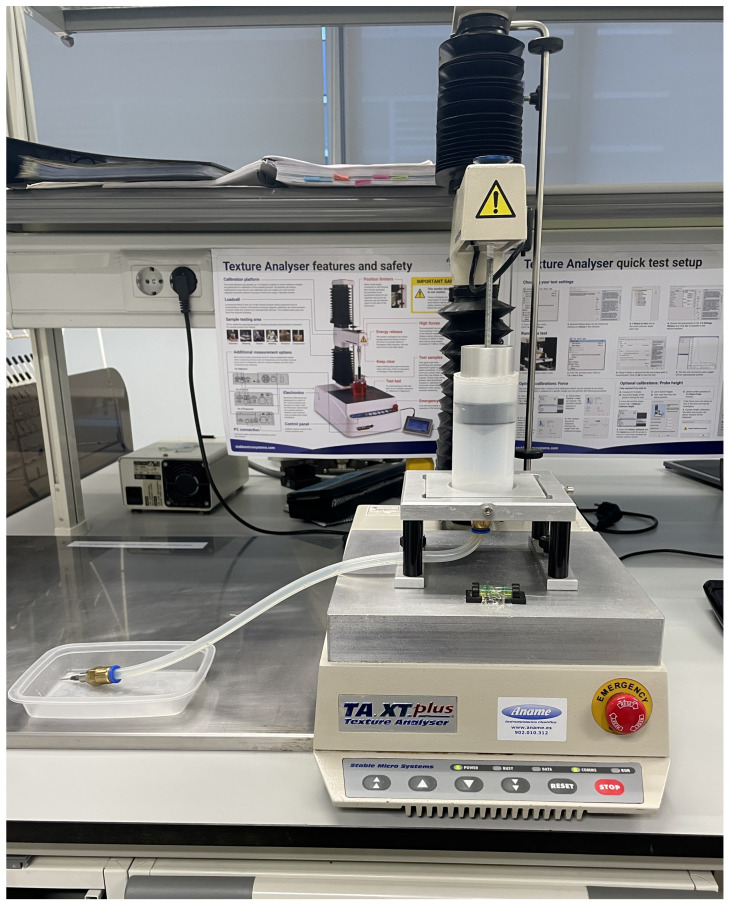
Extrusion test set-up.

The extrusion evaluation of each formulation was analyzed by using a TA.XT.plus texturometer (Stable Micro Systems, Godalming, Surrey, UK) and the Texture Exponent 32 program (Stable Micro Systems, Godalming, Surrey, UK). The extrusion test was designed to simulate the 3D printing process. A syringe (4.95 cm i.d., 1.13 cm i.d. of tip, 10.63 cm length) containing the gel was compressed at 1 mm/s until contact, followed by a slower compression at 0.01 mm/s for 5 mm. The plunger was then held for 300 s to assess relaxation behavior (Figure 1).

The relaxation data were fitted using the linearized Peleg model [31] (Equations (1) and (2)).

(1) $$f\left(t\right)=\frac{F_{0}-F(t)}{F_{0}}$$

(2) $$\frac{t}{f(t)}=\frac{1}{A\cdot B}+\frac{t}{A}$$

where F_0_ is the initial maximum force (N); F(t)(N) is the relaxation force at time t (s); A represents the asymptotic force decay, and B the rate of decay (s^−1^). Parameters were obtained via linear regression.

### 2.4. Figure Design

**Figure 2:**
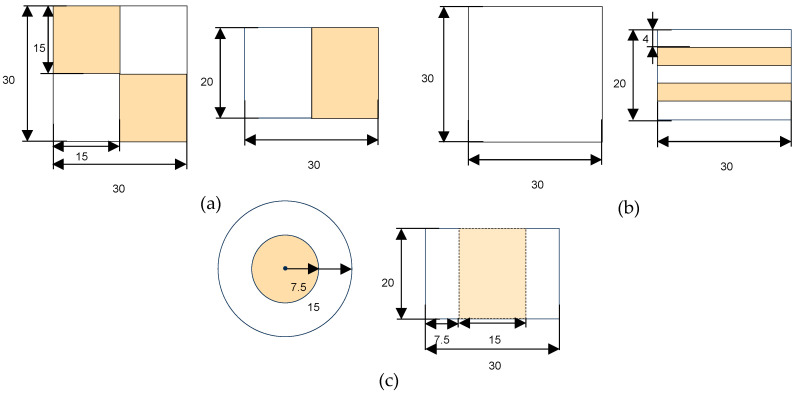
Designed proportions of each of the three shapes in millimeters: (**a**) checkerboard; (**b**) sandwich; (**c**) concentric cylindrical. The white color indicates 0% ink, while the part colored in orange represents edible ink with encapsulated fatty acids.

Three geometries were designed using TinkerCAD (Autodesk Inc., San Francisco, CA, USA): checkerboard (CB), sandwich (SW), and concentric cylinder (CC), each measuring 3 × 3 × 2 cm (Figure 2). The designs incorporated two inks (with and without capsules) in varying proportions to ensure that each printed figure contained at least 3% omega-3, meeting the nutritional claim of being a source of omega-3, as indicated in Regulation (EC) Nº 1924/2006. Given the non-homogeneous distribution of the ink with capsules, the volume of encapsulated ink in the total volume of the figure represented 50% in CB, 40% in SW, and 25% in CC. This is why 0% and 3% of inks were used to print CB, 0% and 3.75% for SW, and 0% and 6% for CC.

### 2.5. Three-Dimensional Printing

**Figure 3:**
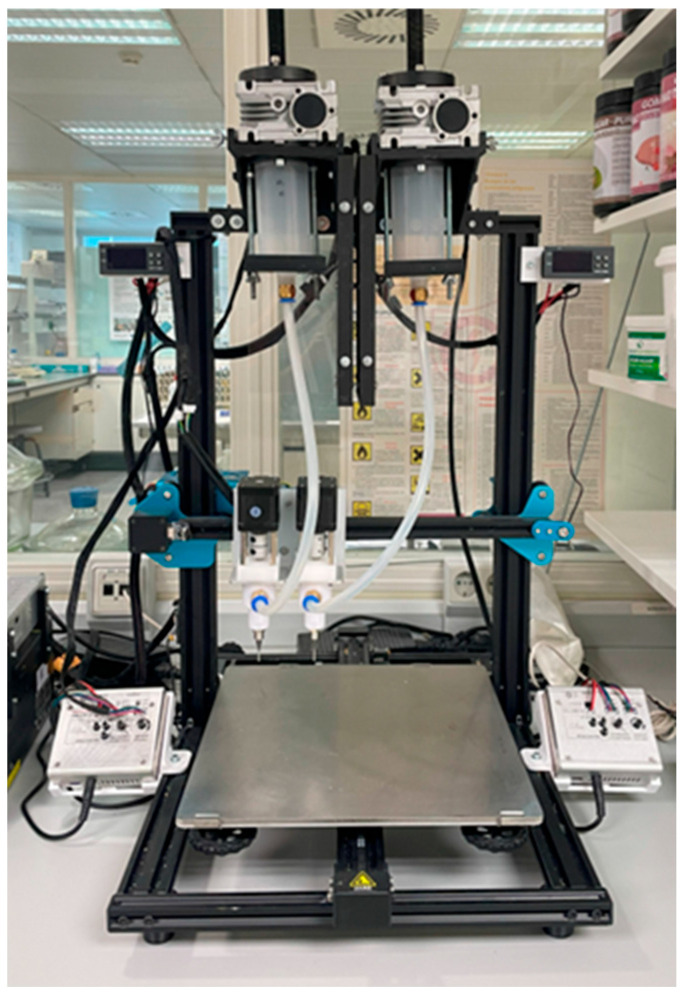
Custom double-head 3D food printer.

**Figure 4:**
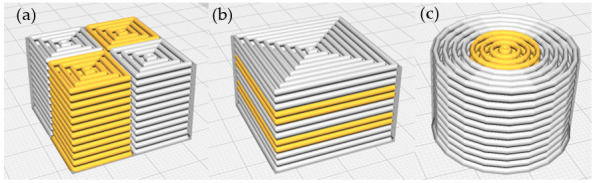
Cura model of (**a**) checkerboard (CB); (**b**) sandwich (SW); (**c**) concentric cylinder (CC). White represents the control formulation (without omega-3 capsules), while orange represents the gel with omega-3 capsules at different concentrations (3% in CB, 3.73% in SW, and 6% in CC).

A custom-built dual-head 3D food printer was utilized for printing assays (Figure 3). The printer consists of two extrusion systems, each composed of a screw system to control the flow of the sample and a smaller one at the tip to facilitate the sample exit. Both systems have an external flow controller, if needed, and another system that controls the actions of the printer. This printer is equipped with two printheads, allowing it to print two different food inks. Preliminary tests were conducted to determine the optimum conditions for each formulation. The printing parameters were standardized across all formulations: flow rate of 10.37 m^3^/s, layer height of 1.2 mm, initial layer height of 1.0 mm, speed of 9 mm/s, and 60% infill. Slicing was performed using UltiMaker Cura (v5.4.0). Finally, the parts of the figure printed with each head were indicated in Cura, distinguishing the two inks used for each sample. (Figure 4). Four replicates of each figure were printed.

### 2.6. Image Analysis

Pictures of printed samples were taken from top and front views at 0, 15, 30, 45, and 60 min post-printing. ImageJ 1.48v software (ImageJ, NIH, Washington, DC, USA) was used to measure dimensional changes over time. Deviations from the original design were calculated to assess print fidelity and structural stability.

### 2.7. Statistical Analysis

All experiments were conducted in triplicate or quadruplicate. Data was analyzed using Statgraphics Centurion 18 (v18.1.13) (Statgraphics Technologies, Inc., The Plains, VA, USA). One-way ANOVA was used to determine significant differences (*p* < 0.05) among formulations. The method used to discriminate between means was Fisher’s least significant difference procedure.

## 3. Results and Discussion

### 3.1. Rheology

**Figure 5:**
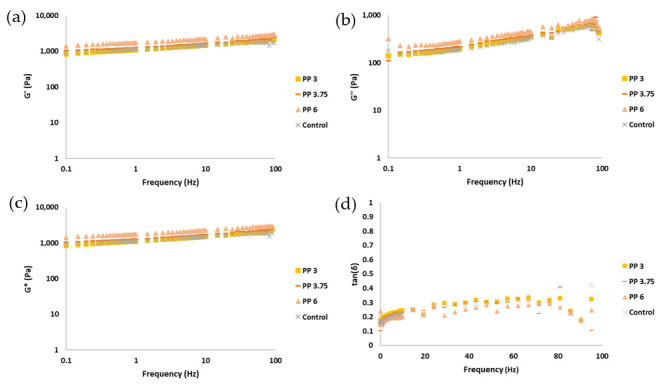
Representation of rheology parameter curves: (**a**) elastic modulus (G′); (**b**) viscous modulus (G″); (**c**) complex modulus (G*); (**d**) tangent phase and apparent viscosity. Samples are coded as pea protein (PP) with the percentage added to that gel (3, 3.75, 6%), and control (without encapsulated omega-3).

One of the key factors that significantly influences extrusion-based 3D printing processes is the rheology of the food ink [32]. The behavior of the flow significantly influences the final structure of the printed material, particularly in terms of its printability. This term includes the ability to pass through a needle (extrudability) and the capacity to maintain the printed form (buildability) [33]. Some of the rheological parameters that predict a good impression include a storage modulus (G′) higher than loss modulus (G″), a low phase angle (tan δ), and intermediate viscosities [28,32,33]. Figure 5 shows oscillatory test parameters, including storage modulus (G′), loss modulus (G″), complex modulus (G*), and phase angle (tan δ) along the frequency sweep.

The rheological behavior of the gel formulations revealed a predominantly elastic character, as indicated by the higher values of the storage modulus (G′) compared to the loss modulus (G″) across the frequency sweep [28]. This viscoelastic solid-like behavior is essential for maintaining the structural fidelity of printed food constructs [28,30,32,34]. The storge modulus (G′) values at 1 Hz were 1107 (115) a, 1101 (55) a, 1266 (99) a, and 1727 (100) b (values indicated as mean, standard deviation between parentheses, and letter of homogeneous group) for control, PP3, PP3.75, and PP6, respectively. Notably, the incorporation of omega-3 capsules led to a progressive increase in G′, with the PP6 formulation exhibiting the highest values and significant differences.

This increase in G′ suggests that the microcapsules act as reinforcing inclusions within the gel matrix, contributing to a more rigid and elastic network. This phenomenon is consistent with findings in other biopolymer systems where particulate fillers or encapsulated bioactives enhance the elastic response by restricting molecular mobility and increasing the number of physical crosslinks [24]. Increases in elastic modulus as well as apparent viscosity are observed in other studies with pea protein and/or inulin for concentrations of 0 to 10% pea protein [35,36]. The increase in storage modulus from incorporating 6% encapsulants, as well as the resulting elasticity of the sample, will impact the force required for printing. To address this increase and enhance printing precision, it is recommended to either increase the ink flow or decrease the printhead speed compared to the control ink. If the capacities of the drive screw or other components are exceeded due to overpressure, the solution would be to increase the internal diameter of the needle. In Figure 5, it can be observed that while G′ increased, the viscous modulus (G″) remained relatively stable or slightly decreased, leading to a lower tan δ (G″/G′ ratio). This indicates a shift toward more solid-like behavior, which is generally favorable for buildability in 3D food printing [33].

Additionally, the phase angle (tan δ) remained below 0.4 for all formulations, indicating a dominant elastic response. This parameter is particularly relevant for predicting buildability, as lower tan δ values are associated with better shape retention post-extrusion [30].

These results suggest that the addition of encapsulated omega-3 does not compromise the rheological properties required for 3D printing. On the contrary, it may enhance the mechanical integrity of the printed structures, particularly at higher concentrations.

### 3.2. Extrusion Process Evaluation

**Figure 6:**
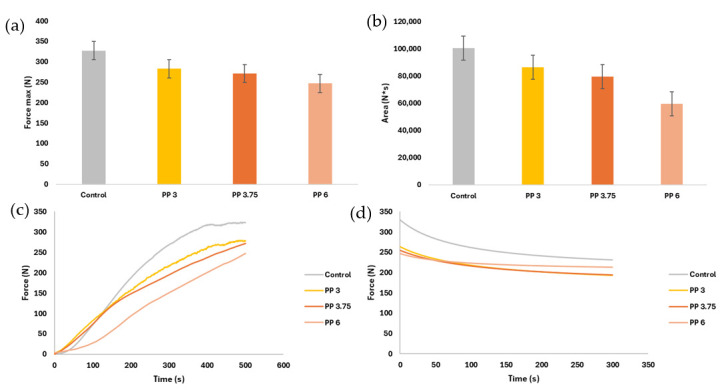
Compression and relaxation test parameters: (**a**) maximum compressive strength; (**b**) area under the compression curve; (**c**) average compression curves; (**d**) average relaxation curves. The intervals displayed show the LSD intervals.

Extrusion tests provided further insights into the mechanical behavior of the gels during the printing process [11,37]. The extrusion test of the samples allows us to know the requirements and structural characteristics when extruded [11,29,37]. With this test, it is possible to know the extrudability of the samples, if they are good for printing, and if the printer used meets the strength requirements. Figure 6 shows the force obtained from the samples over time (c and d) and the comparison between maximum forces and area under the curve (a and b).

For a more direct comparison with the results of other authors, the maximum force was divided by the force contact surface in the test (internal diameter of the syringe). Thus, the results obtained for stress (Pa) were 42,548 (2328) b; 36,737 (5123) a; 35,267 (4462) a; 32,086 (2060) a for Control, PP3, PP3.75, and PP6, respectively. Results are shown as the mean, inter-pair deviation, and the letter of the homogeneous group when performing a simple ANOVA analysis.

Figure 6a shows that the control presented the highest maximum force value. This value has significant differences (*p* < 0.05) with all the samples. In the samples with capsules, no significant differences were observed. As for the area under the curve (Figure 6b), the control sample presented a bigger area, but no significant difference existed between the control and PP 3.

Figure 6c shows the average extrusion test curves for each sample. The control sample presents the highest maximum force, average force, and area under the curve, indicating greater hardness and resistance to flow. On the contrary, the sample with the lowest values of the above parameters was PP6. In this test, the typical curves are composed of two phases. First, when the rod/plunger meets the sample, the force begins to increase with a steep slope. Second, when the sample reaches a maximum or stable value and continues with a stable plateau over time, that represents the average force required to maintain extrusion [38]. These typical extrusion curves can be observed for gels [29,38], gels with fruit pulp [37], white chocolate [39], and various tuber or pumpkin purees [9,40,41]. The samples observed in Figure 6c did not present a slope as steep as in the literature. The steepness of the slope is directly related to the hardness or flexibility of the sample. These samples showed greater flexibility than that observed by other authors since, in most of them, the plateau zone was not present [9,40,41]. The buffer time could be obtained from Control and PP3, i.e., the time required to reach the plateau zone, which indicates the pressure at which the flow is already constant [41]. The control sample had its buffer time at 390 s (6.5 min) and PP3 at around 500 s (8.33 min); the other samples did not reach the plateau zone. The buffer times were excessively large when compared to tubercle and pumpkin samples (25–225 s) [41] or xanthan-konjac gum gels (60–100 s) [38]. High buffer times mean increasing sample flows until a constant force is reached. Although the samples presented extrusion curves with non-constant flows, it does not imply that the figures with these inks present lower stability than those with lower extrusion force or lower buffer times [9]. An effect was observed in the formulation: the greater the number of capsules incorporated, the lower the force to be applied and the longer the buffer time.

The incorporation of encapsulated omega-3 significantly reduced compressive strength, particularly in the PP6 formulation. This reduction in hardness may be attributed to the disruption of the gel network by the microcapsules, which act as inert inclusions and reduce the continuity of the biopolymer matrix. It can be noticed that while the gels with capsules required less force for extrusion, they exhibited longer buffer times—the time needed to reach a steady flow. This suggests a delayed structural rearrangement during extrusion, possibly due to the increased heterogeneity introduced by the capsules. Although these buffer times were longer than those reported for other food matrices (e.g., tuber-based purees [9,11] or xanthan-konjac gels [38]), they did not negatively impact the printability of the samples [37].

Figure 6d shows the relaxation stage of the samples after extrusion. The sample that took the longest time to return to a steady state after stress application was Control, followed by PP3. Table 1 records some of the values of the Peleg model.

The R^2^ values show that the Peleg model fits well for all samples. As for A, the force in so much per one that decays when t tends to infinity, only presented significant differences (*p* < 0.05) when 6% of capsules were incorporated. From the incorporation of 6% of capsules onwards, the samples showed less loss of force when the stress ceased. Finally, no significant differences were found in the speed of force loss when stress ceased. There is no influence of the capsules on the rate of force loss. Similar results of A are shown in Mammarella et al. (2002) [42] for gels of alginate and carrageenan.

Moreover, the presence of capsules increased the buffer time—the time required to reach a steady extrusion flow. This suggests a delayed structural rearrangement during compression, likely due to the heterogeneity introduced by the capsules. Although longer buffer times may be seen as a drawback, they did not negatively impact the printability or stability of the printed structures. The relaxation phase analysis, modeled using Peleg’s equation, showed that samples with higher capsule content had lower force decay (parameter A), indicating better structural recovery after stress. This is advantageous for maintaining the printed shape over time, as it reflects the material’s ability to resist deformation post-extrusion.

Overall, the extrusion results confirm that while the addition of encapsulated omega-3 modifies the mechanical properties of the gels, it does not hinder their suitability for 3D printing. The improved recovery and reduced extrusion force may facilitate printing with lower mechanical demands, broadening the potential for application in consumer-grade printers. Comparing both textural and rheological analysis, it can be observed that while the incorporation of encapsulated omega-3 increased the elastic modulus (G′), it simultaneously reduced the maximum compression force. This apparent contradiction can be explained by the dual role of the microcapsules. On one hand, they act as rigid inclusions that locally reinforce the gel matrix, enhancing its elastic response under small deformations (as in oscillatory tests). On the other hand, they disrupt the continuity of the gelatin–carrageenan network, reducing the overall cohesion and resistance to sustained compressive stress. This leads to localized stress concentrations and easier structural collapse, resulting in lower extrusion force requirements. Similar behavior has been reported in composite gels where fillers increase stiffness but decrease compressive strength due to network discontinuities and stress concentration points [24,38]. Additionally, the capsules may facilitate internal slippage or act as lubricants, further lowering the extrusion force required. Finally, despite the comparison of these two analyses, it is worth mentioning that each test is performed under different measurement conditions, so they would not be very comparable. While rheology is measured under conditions of no permanent deformation, extrusion presents forces where the sample is permanently deformed [43].

### 3.3. Image Analysis

The image analysis provided valuable insights into the buildability and dimensional stability of the printed structures over time. By comparing the printed figures at time 0 and after 60 min, it was possible to assess both the initial print fidelity and the structural integrity during storage.

**Figure 7:**
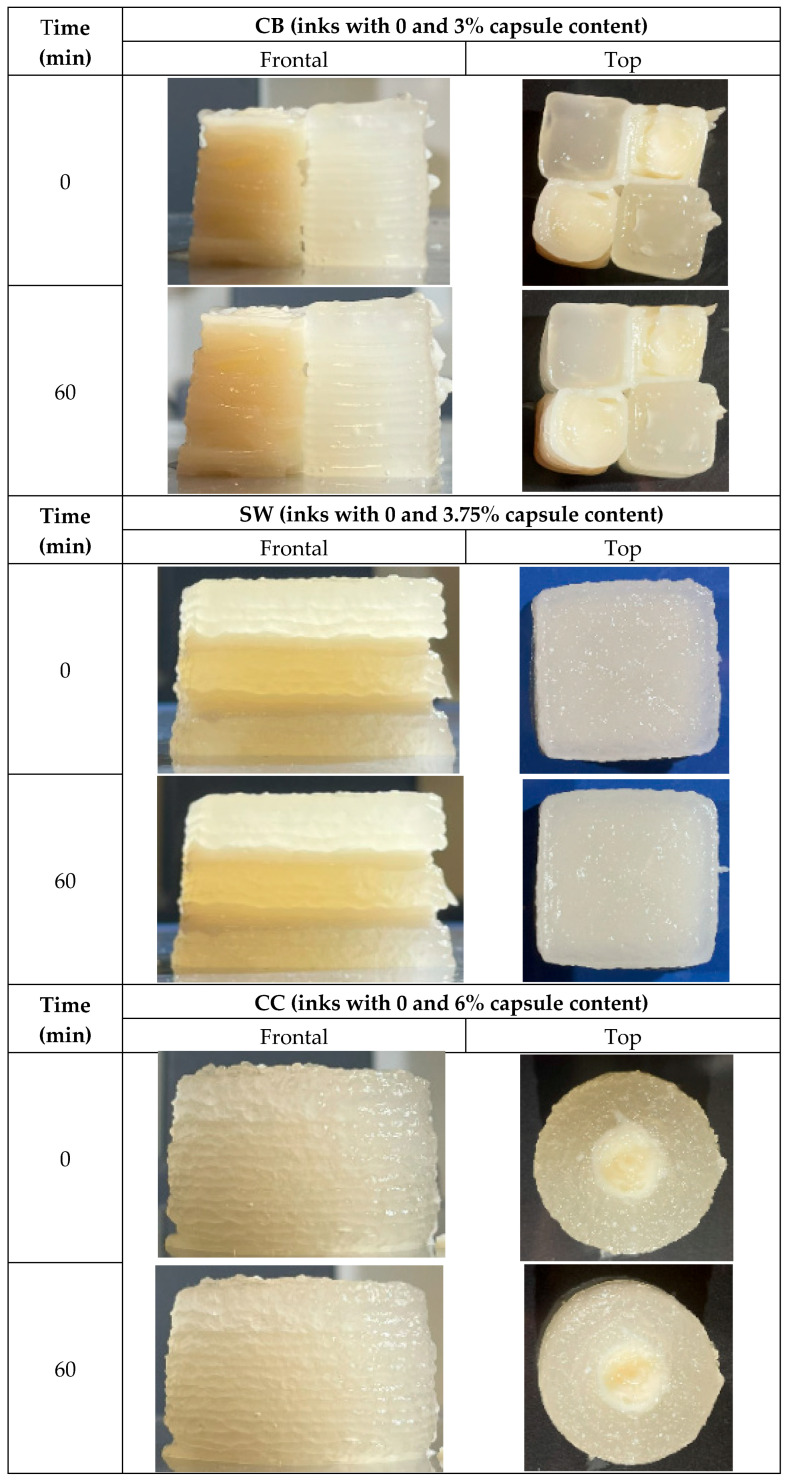
Three-dimensionally printed samples front and top views, time 0 printed and after 60 min. (CB: checkerboard; SW: sandwich; CC: concentric cylinder).

Image analysis makes it possible to assess the buildability of the ink, i.e., the part of printability that refers to whether a material is good at preserving its shape over time. This is why pictures are usually taken over time, up to 60 min, to compare with the original at time 0. On the other hand, by analyzing the time 0 images against the proportions of the designed figure, conclusions can be drawn about part of its extrudability. Extrudability refers to whether a material is good for printing (extrusion process). Figure 7 shows the printed samples at times 0 and 60 min later. Table 2 shows the deviation values in percentage of the proportions of the figure at time 0 concerning the designed proportions.

A noteworthy aspect that has not been assessed in the study is that the incorporation of the encapsulants modifies the color of the base formulation to whiter, yellowish tones (Figure 7). Dimension by dimension, CB was the sample with the closest height to the one that was originally designed. Regarding figure width, SW was the one with the most significant difference. In the diameter/depth deviation, all samples present a similar deviation; CB was the one with negative values, which means the figure had a shallower depth than the one designed. Finally, in the area deviation, it was observed that the SW sample had a minor deviation from the others, CC had a high positive deviation (the sample had a larger area than the designed one), but CB was the only one with a smaller area than the designed one.

**Figure 8:**
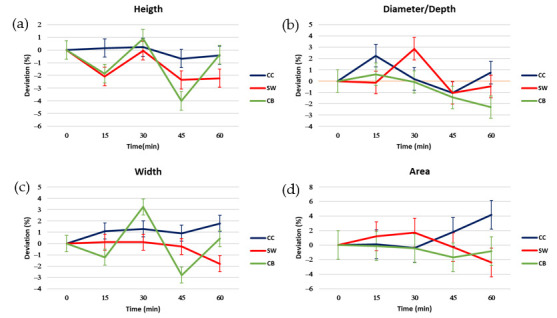
Deviations in percentage of the proportions of the figures with time (0–60 min) with respect to the time 0 min: (**a**) height, (**b**) width, (**c**) diameter, (**d**) area. The LSD intervals of each proportion are represented in the graphs.

To follow the evolution of each proportion of the samples, the measurements of the proportions at time 0 were taken as a reference for each time. Figure 8 shows the evolution of each proportion over time for each sample by the deviation (%) of the respective time from time 0.

Observing the height graph (Figure 8a), the samples showed significant differences between them at 15, 45, and 60 min. The sample that showed the greatest stability in terms of height was the CC sample.

Regarding width, the CB sample showed the most considerable deviations over time. SW and CC presented a less pronounced deviation over time, being the time 60 min where the biggest deviation occurring at 60 min. Also, at 60 min, CC and SW had significant differences; in the case of CC, it was due to their ending with an average value of +2, and SW with an average value of −2.

In the diameter or depth graph, the sample that was more stable on average was CB. In this case, the CC sample showed greater stability variation, but no significant differences were observed with CB (the most stable on average) except for the time of 60 min.

Finally, regarding area deviations, the most stable sample on average was CB (3% capsules). It should be noted that the only significant difference between the samples was in the 60 min time between the CC (6% capsules) sample and the SW (3.75% capsules) and CB samples. Observing the significant differences and the average deviations, the CC sample would be the most stable over time, with the area of the upper perspective being the proportion with the biggest deviation.

Among the three geometries tested—checkerboard (CB), sandwich (SW), and concentric cylinder (CC)—the CB configuration exhibited the lowest deviation from the designed dimensions at time 0, particularly in height and area. This suggests that the CB design offers superior extrudability, likely due to its more uniform distribution of inks and reduced structural complexity. In contrast, the CC figure showed the highest area deviation, indicating over-extrusion or spreading, which may be attributed to the radial distribution of inks and the higher proportion of encapsulated material.

Over time, the CC configuration demonstrated the greatest stability in height, while the CB figure was the most stable in terms of area. These findings highlight a trade-off between initial fidelity and long-term stability, which is influenced by both the geometry and the formulation used. The SW figure, although intermediate in most parameters, showed the least deviation in area at time 0, suggesting a balanced performance.

The results align with previous studies that emphasize the importance of ink rheology and structural design in determining print quality and stability [30,37]. The presence of encapsulated omega-3 appears to enhance structural integrity over time, particularly in the CC (6% PP) configuration, possibly due to the increased elastic modulus observed in rheological tests. However, this comes at the cost of reduced print fidelity, especially at higher capsule concentrations.

These observations underscore the need to optimize both formulation and geometry when designing functional 3D-printed foods. Future work could explore the use of predictive models to correlate rheological parameters with print outcomes, as well as the role of capsule size and distribution in structural performance.

## 4. Conclusions

The incorporation of omega-3 capsules encapsulated in pea protein into a gelatin–carrageenan gel matrix significantly influenced the rheological, textural, and printability properties of the formulations when it was incorporated at 6%. Rheological analysis revealed an increase in elastic behavior with capsule concentration, while extrusion analysis showed a reduction in gel hardness and an increase in buffer time during extrusion. These changes did not compromise the printability of the inks but rather highlighted the need to balance formulation and design. Despite finding significant differences between the formulations, all samples were able to print and maintain a stable shape, so that a maximum force range between 225 and 350 N or 32,000 and 42,500 Pa of stress and elastic modulus values at 1 Hz between 1101 and 1727 Pa are good for printing. Future studies using models such as Herschel–Bulkley could further investigate the behavior of the different formulations.

Among the three geometries tested, the checkerboard (CB) configuration demonstrated the best overall performance, with the highest print fidelity and the lowest dimensional deviation fresh printed. Although the concentric cylinder (CC) figure showed superior height stability, it suffered from significant over-extrusion and area deviation over time. The sandwich (SW) design exhibited intermediate behavior.

Despite not carrying out a study where the formulations and figures are intertwined, the CB geometry is recommended for future applications where both visual accuracy and structural stability are critical. These findings support the feasibility of using encapsulated bioactive compounds in 3D food printing to develop functional foods with tailored mechanical and nutritional properties.

## Figures and Tables

**Table 1:** Parameters of the Peleg model fit the average relaxation curves.

Sample	R^2^	A	B (s^−1^)
Control	0.95 (0.05) ^a^	0.38 (0.02) ^b^	0.012 (0.002) ^a^
PP3	0.95 (0.05) ^a^	0.37 (0.07) ^b^	0.009 (0.004) ^a^
PP3.75	0.93 (0.03) ^a^	0.33 (0.03) ^b^	0.0084 (0.0011) ^a^
PP6	0.96 (0.03) ^a^	0.18 (0.06) ^a^	0.015 (0.007) ^a^

The results are expressed as mean and standard deviation in parentheses. Letters in each column (a,b) refer to homogeneous groups according to ANOVA (*p* < 0.05).

**Table 2:** Deviation in percentage of the proportions of the newly printed figures vs. the designed ones.

Sample	Height Deviation (%)	Width Deviation (%)	Diameter/Depth Deviation (%)	Area Deviation (%)
CC	9(3) ^b^	2(4) ^a^	7(16) ^b^	21(10) ^c^
SW	10(3) ^b^	8(4) ^b^	5(3) ^b^	4(4) ^b^
CB	3(7) ^a^	2(5) ^a^	−4(6) ^a^	−12(7) ^a^

The results are expressed as mean and standard deviation in parentheses. Letters in each column (a–c) refer to homogeneous groups according to ANOVA (*p* < 0.05).

## Data Availability

The original contributions presented in the study are included in the article, further inquiries can be directed to the corresponding author.
